# Relationships between *GAT1* and PTSD, Depression, and Substance Use Disorder

**DOI:** 10.3390/brainsci7010006

**Published:** 2017-01-05

**Authors:** Kaitlin E. Bountress, Wei Wei, Christina Sheerin, Dongjun Chung, Ananda B. Amstadter, Howard Mandel, Zhewu Wang

**Affiliations:** 1 Department of Psychiatry, Medical University of South Carolina, Charleston, SC 29425, USA; bountres@musc.edu; 2 Department of Public Health Sciences, Medical University of South Carolina, Charleston, SC 29425-8350, USA; weiwe@musc.edu (W.W.); chungd@musc.edu (D.C.); 3 Virginia Institute for Psychiatry and Behavioral Genetics, Virginia Commonwealth University, Richmond, VA 23219-1534, USA; Christina.sheerin@gmail.com (C.S.); Ananda.Amstadter@vcuhealth.org (A.B.A.); 4 Ralph H. Johnson VA Medical Center, Charleston, SC 29401, USA; mandel@musc.edu

**Keywords:** *GAT1*, post-traumatic stress disorder, major depressive disorder, substance use disorder, veterans

## Abstract

Post-traumatic stress disorder (PTSD), Major Depressive Disorder (MDD), and Substance Use Disorder (SUD) have large public health impacts. Therefore, researchers have attempted to identify those at greatest risk for these phenotypes. PTSD, MDD, and SUD are in part genetically influenced. Additionally, genes in the glutamate and gamma-aminobutyric acid (GABA) system are implicated in the encoding of emotional and fear memories, and thus may impact these phenotypes. The current study examined the associations of single nucleotide polymorphisms in *GAT1* individually, and at the gene level, using a principal components (PC) approach, with PTSD, PTSD comorbid with MDD, and PTSD comorbid with SUD in 486 combat-exposed veterans.  Findings indicate that several *GAT1* SNPs, as well as one of the *GAT1* PCs, was associated with PTSD, with and without MDD and SUD comorbidity. The present study findings provide initial insights into one pathway by which shared genetic risk influences PTSD-MDD and PTSD-SUD comorbidities, and thus identify a high-risk group (based on genotype) on whom prevention and intervention efforts should be focused.

## 1. Introduction

There exists a notable, and important, disconnect between rates of traumatic event exposure and development of negative outcomes such as post-traumatic stress disorder (PTSD). Given the fact that only a subset of individuals go on to develop the intense and prolonged stress response characterized by PTSD, great interest exists in identifying individual differences that confer this risk. Among those risk factors of interest, individual genetic differences represent an important etiologic source of variability in risk, supported by extant lines of evidence from family, twin, and molecular genetic studies [1].

The molecular genetics literature on PTSD, which seeks to identify specific genetic variation that may account for increased risk for the phenotype given trauma exposure, has grown substantially over the last two decades, with over 120 molecular genetic associations published to date (see [2,3] for reviews). Although genome-wide association studies that agnostically examine polymorphisms across the entire genome are increasing in frequency, the vast majority of extant molecular genetic studies have utilized a candidate gene design. In this design, selection of the gene(s) is informed by existing biological evidence, targeting polymorphisms (i.e., genetic variation) within and around the gene region of interest. Genes examined from biological candidate systems in relation to PTSD have included polymorphisms of genes from the dopaminergic system (e.g., DAT1, *DRD2* [4]), the promoter region of the serotonin transporter system (*5-HTTLPR* [5]), and the hypothalamic pituitary adrenal axis (i.e., stress-response) system (e.g., glucocorticoid receptor [6]), among others. These studies have met with varying success and there is much still unknown about the molecular genetic architecture of PTSD.

### 1.1. Glutamate/GABA Systems and Risk for PTSD

More recently, there has been increased interest in examining genes related to the glutamate and γ-aminobutyric acid (GABA) systems in informing our understanding of the genetic underpinnings of PTSD [7]. Existing preclinical animal studies support the notion that the GABAergic system regulates the intensity and duration of the stress response and plays a role in controlling the epigenetic and gene transcriptional responses to psychological stress (see [8]) as well as being involved in the encoding of emotional and fear memories [9,10].

Under the assumption that GABA levels in the brain are genetically predetermined, findings of differential GABA levels in individuals with PTSD suggest that this may represent preexisting vulnerability to stress-related disorders such as PTSD [11]. Thus, examination of genetic variation within this gene and its association with PTSD would further our understanding of the GABAergic system in relation to PTSD as well as our understanding of genetic risk of this system as a vulnerability marker for PTSD. Within the GABAergic system, the GABA transporters (GATs) are key molecules in GABAergic transmission. GATs control the duration and intensity of GABAergic activity at the synapse, through reuptake of released GABA [12]. There are four GABA transporter subtypes, GAT1, GAT2, GAT3, and GAT4, with *GAT1* coding for *GAT1*, which is considered the major subtype and presents both at synaptic and extrasynaptic sites in the brain [13,14]. Variation in the *GAT1* gene may play a part in impacting predisposition to negative outcomes following trauma, which has been supported in animal models of anxiety [12] and learning/memory (e.g., [15]). This work is clearly still in its infancy, as a review of the literature suggests that, to date, there have been no clinical investigations of the association between *GAT1* and PTSD.

### 1.2. The GABA System and Other Psychiatric Comorbidities

The extant literature suggests that much of the demonstrated genetic influences on PTSD overlap with those of other conditions, particularly depression [16]. These shared genetic influences may underlie the frequent comorbid presentation of these disorders. Given the role of the GABA system in the brain and in response to stressors, as well as associations of GABA activity in a range of emotional and cognitive factors, it is unsurprising that variation in this system has been suggested as a potential marker that may cut across clinical diagnostic categories [15]. In particular, GABA abnormalities have been associated with Major Depressive Disorder (MDD; e.g., [17,18,19]). If the GABA system is involved in the encoding of emotional and fear memories [9,10], individuals at greater genetic risk on *GAT1* may show increased propensity for comorbid PTSD-MDD, rather than just PTSD alone.

In addition to the high degree of comorbidity between PTSD and MDD, PTSD and substance use disorders (SUDs) also frequently co-occur [20,21,22]. The extant literature suggests that many of the demonstrated genetic influences on PTSD overlap with those of alcohol [23,24] and drug use disorders [25]. Additionally, many individuals with PTSD use alcohol and drugs in order to avoid aversive emotional symptoms (i.e., “self-medication” [26]). Given GABA’s role in regulating stress response [8] and encoding emotional and fear memories [9,10], it may be that variation in *GAT1* represents a potential shared genetic risk for both greater trauma-related anxiety (i.e., greater severity of PTSD symptoms) and use/misuse of substances following trauma exposure. Because of the paucity of work on GABA system genes and SUD, we will examine *GAT1*’s impact on PTSD-SUD comorbidity in an exploratory manner.

### 1.3. Current Study

The first aim of the present investigation was to examine the association between *GAT1* with PTSD in a trauma-exposed sample. Specifically, the current study tests the hypothesis that *GAT1* variation, utilizing both single nucleotide polymorphisms (SNPs) and principle components comprised of these polymorphisms, will significantly predict PTSD diagnosis. The second aim of this study is to examine whether *GAT1* variation underlies risk for comorbid PTSD and MDD. It is hypothesized that *GAT1* will be significantly associated with comorbid PTSD-MDD diagnosis, such that those at higher genetic risk will be more likely to meet criteria for both PTSD and MDD, rather than just PTSD alone. The final (and exploratory) aim of this study is to examine whether SNPs within the *GAT1* gene underlie increased risk for comorbid PTSD and SUDs, over and above the risk for PTSD alone. Specifically, the current study tests whether individuals at higher genetic risk on *GAT1* are more likely to meet criteria for PTSD and SUD, compared to PTSD alone. This project aims to expand upon existing work through the use of multiple SNPs and principle components as measures of genetic risk. It also examines whether these genetic effects influence PTSD, PTSD and MDD, or PTSD and SUD, over and above the covariates with established impact on outcomes including age, sex, race, and combat exposure severity.

## 2. Materials and Methods

### 2.1. Participants

A total of 519 combat veterans, recruited through the Cincinnati Veterans Affairs Medical Center (VAMC) and Charleston VAMC, were enrolled in the study. Participants were eligible for inclusion if they had a history of combat exposure, and if they did not meet criteria for lifetime DSM-IV schizophrenia, bipolar disorder, or active substance abuse or dependence in the past six months. Of the 519 enrolled, genotype data were missing for 33 individuals, who were thus excluded. The 486 remaining participants were included in study analyses. Of these 486, 82% were male, 69% were Caucasian, and all were between the ages of 21–80. All 486 were in the control or PTSD group, all of whom experienced combat exposure. Attempts were made to match the trauma-exposure severity between these two groups. Specifically, all control participants from the Charleston VAMC and a majority of control participants from the Cincinnati VA were required to have Combat Exposure Scale (CES) scores of 10 or more. All control subjects were also selected to be free of Axis I disorders. Additional information about control and PTSD study participants has been previously reported [27].

### 2.2. Procedure

All subjects gave their informed consent for inclusion before they participated in the study. The study was conducted in accordance with the Declaration of Helsinki, and the research protocol was approved by the Institutional Review Boards (IRB) of both the University of Cincinnati and Medical University of South Carolina. Demographic and deployment information was collected after participants provided consent. PTSD symptoms, as well as other major psychiatric disorders, were assessed by a board-certified psychiatrist. Additional interview and self-report measures were implemented to capture additional information (e.g., combat exposure information).

### 2.3. Measures

See Table 1 for descriptive information on study constructs.

#### 2.3.1. Age, Sex, and Race

Because the relationships among study variables tend to vary across age, sex, and race [28,29,30], these constructs were used as covariates in study models. Specifically, individuals self-reported on age in years, sex, and race. In order to examine the effects of race on study outcomes, two dummy-coded variables were created. The first compared Caucasians to African-Americans, and the second compared Caucasians to those who self-identified as a race other than Caucasian or African-American.

#### 2.3.2. Combat Exposure Scale

The Combat Exposure Scale (CES; [31]) is a seven-item self-report measure used to obtain information regarding exposure to wartime stressful events. The measure has total scores ranging from 1 to 41, with a higher number reflecting higher severity of combat exposure.

#### 2.3.3. Post-Traumatic Stress Disorder

Participants were interviewed using the clinician administered PTSD Scale (CAPS [32]), a structured diagnostic interview for current and lifetime PTSD that assesses all 17 symptoms of PTSD for frequency and intensity. Those who endorsed at least one re-experiencing symptom, three or more avoidance symptoms, and two or more hyperarousal symptoms met the criteria for DSM-IV PTSD.

#### 2.3.4. Depression

Participants were interviewed using the Hamilton Rating Scale for Depression (Ham-D [33]), a 17-item semi-structured interview for current Depression. Individuals who received scores of 20 or higher met criteria were considered to meet criteria for depression.

#### 2.3.5. Substance Use Disorder

Participants were interviewed about their lifetime substance use using by a board-certified psychiatrist with either the Structured Clinical Interview for DSM-IV (SCID [34]) or the Mini-International Neuropsychiatric Interview (MINI [35]).

#### 2.3.6. SNP Selection and Genotyping

Genomic DNA was extracted from blood samples using a Wizard Genomic DNA purification kit (Promega, Madison, WI, USA) using the manufacturer’s protocol. Sixteen SNPs thought to be representative in terms of coverage of the entire *GAT1* gene, as well as those that were not in complete linkage disequilibrium with others within the gene, were included. Additionally, variants with minor allele frequencies greater than or equal to 10%, and covering a region of 50 kb in the *NET* gene were included (upstream promoter region of *NET* gene and spanning 46 kB toward the 3′ end of the *SLC1A1* gene using a software tool (SNPbrowser version 4.0; Applied Biosystems, Foster City, CA, USA). Additional information on genotyping can be found in author et al. [27]. Table 2 provides a summary of the 16 SNPs examined in this study including their genotype frequencies and *p*-values for testing Hardy–Weinberg Equilibrium (HWE) by race. No violation of HWE assumptions were detected after the Bonferroni correction. Table 2 also lists the *p*-values for comparing the genotype frequencies between African-Americans and Caucasians.

### 2.4. Data Analytic Plan

A preliminary analysis was conducted to examine the differences between the control group and PTSD cases in terms of demographic information (age, sex, and race), as well as the association with MDD, SUD, and amount of combat exposure (via the CES). T-tests and chi-square tests were used for preliminary analysis. The primary analysis assessed the risk of three different outcomes: the presence of PTSD (i.e., including all of PTSD without MDD/SUD, PTSD with MDD, and PTSD with SUD), the comorbidity of PTSD and MDD, and the comorbidity of PTSD and SUD. The association between each SNP and PTSD was analyzed using logistic regression after adjusting for the effects of age, sex, race, and CES. Proportional odds models were used to study the association between each SNP and each of the comorbidities after adjusting for demographic variables and CES. The key assumption of the proportional odds model is that the coefficients that describe the relation between the categories lower than or equivalent to a certain category versus the categories higher than this category in the response variable are the same for all categories in the response variable [36]. Our proportional odds models predict membership in the ordered categories: no PTSD, PTSD without comorbidities, and PTSD with comorbidities.

After we fitted the model for each of 16 SNPs, a Bonferroni correction was employed to adjust their PTSD association *p*-values for multiple testing. To control for familywise type I error rate, we adjusted p values using the Bonferroni approach for the 16 SNPs but not for the three outcomes, because the Bonferroni adjustment is known to be somewhat conservative, especially when the tests are correlated [37]. Based on this procedure, and given the significance level of α = 0.05 for each SNP, we determined a SNP to be associated with each outcome if *p* < 0.003.

Given the limited sample size in our study, it was possible that we were under powered to reach significance. To address this issue, we also considered a gene-based association analysis that improves statistical power by sharing information across multiple SNPs. For this gene-based analysis, we employed a principal component (PC) approach that has been shown to be superior than the haplotype based approaches in terms of having better power to identify gene-disease association and producing less biased estimates [38]*.* For this approach, we constructed PCs using the allele counts of all 16 SNPs after standardizing allele counts of each SNP, and included the first several PCs with the largest variances in the model to represent the gene.

For these analyses, we included only Caucasian participants. This decision was made because the genotype frequencies were significantly different between African-Americans and Caucasians as shown in Table 2. Additionally, because African-Americans only made up 24.3% of the study cohort, the analyses on this small group of participants may have been underpowered and/or the estimates may not be stable or representative of the larger group of African-Americans.

Within these analyses on Caucasians, we considered the first four principal components (PC1, PC2, PC3, and PC4) in our analysis because they explained 57% of genotype variance (i.e., 19%, 15%, 13%, and 10%, respectively). (The fifth and sixth components explained 8% and 6% of the variance, respectively. We chose the number of components by plotting the percent variance associated with each PC). The PC approach is not only known to effectively capture the LD information within a gene, but it is also easy to implement, and is reported to be more powerful than other genotype- or haplotype-based approaches such as the ranked sum score approach [36,37]. The association between the first four principal components and each of the three outcomes was assessed using logistic regression or proportional odds logistic regression, similar to those described above.

## 3. Results

In terms of demographic and clinical descriptive comparisons, there were no statistically significant differences between PTSD cases and controls with regard to age (*p* = 0.820). However, CES (*p* = 0.020) and race (*p* = 0.001) were found to be predictors of PTSD status, with sex being a marginally significant predictor (*p* = 0.056). Specifically, subjects with higher CES, African-Americans, and males were at higher risk for PTSD. MDD (*p* < 0.001) and SUD (*p* < 0.001) were predictive of PTSD. Out of the 314 PTSD cases, 203 (64.6%) cases were also diagnosed with MDD, 99 (31.5%) cases had a history of SUD, and 78 (24.8%) cases had PTSD without MDD or SUD, whereas only six out of the 172 controls (3.5%) had MDD or SUD.

### 3.1. SNP Level Association

Table 3 summarizes the effect of each SNP on risk for PTSD, the comorbidity of PTSD and MDD, and the comorbidity of PTSD and SUD, respectively. Although the analyses presented in Table 3 pertain to the whole sample, results of Caucasian-specific and African-American-specific analyses can be viewed in Appendix Table A1 and Table A2, respectively. The PTSD analysis results based on logistic regression analysis indicate that the minor allele of rs720352 (OR = 1.35, 95% CI: 1.01–1.81, *p* = 0.045) and rs2272403 (OR = 1.45, 95% CI: 1.01–2.10, *p* = 0.046) increased the risk of PTSD, whereas the minor allele of rs1919075 (OR = 0.65, 95% CI: 0.47–0.89, *p* = 0.008) reduced the risk of PTSD, after adjusting for age, race, sex, and CES, although these results did not remain significant after the Bonferroni correction for multiple testing. In the comorbidity analysis for PTSD and MDD based on the proportional odds model, the minor allele of rs1919075 also reduced the odds of having PTSD or PTSD-MDD (OR = 0.71, 95% CI: 0.53–0.94, *p* = 0.018), adjusting for age, race, sex and CES. Specifically, for patients with the minor allele of rs1919075, their odds of having PTSD-MDD versus having PTSD alone or no PTSD are 29% lower compared to patients without this minor allele given age, race, sex and CES are held constant. Likewise, their odds of having PTSD or PTSD-MDD versus no PTSD is 29% lower compared to patients without this minor allele, given other variables in the model are held constant.

The minor allele of rs2272403 (OR = 1.44, 95% CI: 1.07–1.95, *p* = 0.018) significantly increased the odds of having PTSD or PTSD-MDD, with all the other variables in the model held constant. In contrast, the minor allele of rs2928078 (OR = 1.32, 95% CI: 1.02–1.71, *p* = 0.033) and rs2933308 (OR = 1.33, 95% CI: 1.03–1.73, *p* = 0.028) significantly increased the odds of having PTSD or PTSD-SUD, adjusting for age, race, sex, and CES, whereas the minor allele of rs1919075 reduced the odds of having PTSD or PTSD-SUD (OR = 0.66, 95% CI: 0.49-0.89, *p* = 0.006). These findings were also not significant after Bonferroni correction (*p* < 0.003).

The association between each of the 16 SNPs and the three outcomes (PTSD, PTSD-MDD, and PTSD-SUD) were also examined by race. Similar findings were obtained for Caucasians, whereas a smaller number of SNPs significantly predicted these outcomes among African-Americans.

### 3.2. Gene Level Association

Interrogation of the loading matrix (Table 4) indicated that PC1 is mostly associated with rs2928078, rs2933308, rs720352, and rs1919075, which is consistent with LD structure among Caucasians. These SNPs are predictive of PTSD, PTSD-MDD, or PTSD-SUD. Specifically, PC1 was inversely associated with rs2928078 (ρ = −0.51), rs2933308 (ρ = −0.46), rs720352 (ρ = −0.46), and positively associated with rs1919075 (ρ = 0.39). Hence, the association between PC1 and outcomes of interest found in Caucasians represents the overall effect of SNPs located at *GAT1* gene on PTSD, PTSD-MDD, and PTSD-SUD, respectively.

Table 5 shows the gene-level analysis results based on the PC approach. Among the four principal components we considered, PC1 was significantly associated with PTSD (OR = 0.84, 95% CI: 0.73–0.96, *p* = 0.012) among Caucasians, after adjusting for age, sex, and CES. Based on the proportional odds model, PC1 was also significantly associated with risk of PTSD or PTSD-MDD (OR = 0.88, 95% CI: 0.78–0.99, *p* = 0.033) after adjusting for age, sex and CES. Similarly, PC1 was significantly associated with the risk of PTSD or PTSD-SUD (OR = 0.86, 95% CI: 0.76–0.97, *p* = 0.015) after adjusting for age, sex, and CES. Age, sex, and CES were not significantly associated with any of the three outcomes. (To verify the validity of our PCA approach, we additionally considered the genetic risk sum score (GRSS) [39] to examine the gene level association with phenotypes. Specifically, GRSS is calculated by the summation of the number of risk alleles across SNPs divided by the number of SNPs in the score, where risk alleles are determined based on *p*-values after filtering out highly correlated SNPs. We calculated GRSS based on a *p*-value cutoff point of 0.05, and included the GRSS as a predictor in our multivariate logistic and proportional odds models. We found that a higher number of risk alleles is associated with increased risk of PTSD, PTSD-MDD, and PTSD-SUD (see Appendix Table A3).) As with the previous analyses initially found to be significant (*p* < 0.05), these results were not significant after Bonferroni correction (*p* < 0.003).

## 4. Discussion

The current study had three aims. First, we examined whether *GAT1* variation significantly predicted PTSD diagnosis. Second, we tested the hypothesis that *GAT1* would be significantly associated with comorbid PTSD-MDD diagnoses. Finally, we investigated whether individuals at higher genetic risk on *GAT1* would be more likely to meet the criteria for PTSD and SUD. Each of these aims will be discussed in turn.

In examining the relationships between single SNPs and each of the PCs and PTSD, we found preliminary evidence that PC1 was significantly associated with PTSD diagnosis. These findings are consistent with research suggesting that *GAT1* may impact anxious behaviors [12,13] and fear extinction [9] in rats. The findings also add to the limited work examining the impact of transporters in the GABA and glutamate systems on PTSD diagnosis in veterans (i.e., the SNP rs10739062 predicted PTSD diagnosis; [27]). Thus, the current study findings provide preliminary support for the impact of *GAT1* on emotion/fear-based memories in veterans.

In testing the second study hypothesis, PC1 was associated with increased risk for comorbid PTSD and MDD diagnoses, over PTSD without MDD. This finding is consistent with prior animal work suggesting that *GAT1* may impact both anxiety and depression-like behaviors [12,19]. However, this study is the first to find that these SNPs in particular may impact PTSD-MDD comorbidity. We also add to the literature by suggesting that *GAT1* underlies some shared generalized risk (e.g., altered mood/fear-based behavior post-trauma) that is associated with risk for both PTSD and MDD. Future work should seek to identify specifics of this shared risk and endophenotypes, with the aim of pinpointing one target for interventions to prevent both PTSD and MDD post-trauma.

In terms of the final and exploratory aim, PC1 predicted increased propensity for comorbid PTSD and SUD diagnoses, over PTSD without SUD, over and above study covariates including severity of trauma exposure. This finding is consistent with the larger literature implicating shared genetic risk in propensity for this PTSD-SUD comorbidity [24]. However, these findings are the first to find that some of these *GAT1* SNPs impact PTSD-SUD comorbidity. They also add to this work by identifying *GAT1* as one mechanism by which risk for both PTSD and SUD is conferred.

We also examined the relationship between 16 SNPs and our three outcomes (PTSD, PTSD-MDD, and PTSD-SUD). None of these associations reached our a priori significance threshold.

### Strengths and Limitations

Despite the current study’s strengths, including the use of a powerful PC approach to capturing genetic risk, it is important to acknowledge its limitations. First, our data are cross-sectional. Therefore, the temporal associations among these phenotypes are unclear. Specifically, individuals at higher risk on *GAT1* may have been more likely to experience PTSD post-trauma, and then subsequently develop MDD and/or SUD. Moreover, SUD was, by study design, only lifetime and not current. Alternatively, individuals at higher risk on *GAT1* may have been more likely to meet the criteria for MDD or SUD, in turn increasing their vulnerability to PTSD post-trauma. More research is needed to test more specifically how *GAT1* might influence these comorbidities. Additionally, the current study findings cannot rule out the possibility that some unmeasured confounder (e.g., potentially another gene that is associated with *GAT1*) explains the risk for PTSD-MDD or PTSD-SUD. The findings also cannot rule out the possibility that the effect of PC1 is being driven by a single SNP. Finally, without including ancestry informative markers, population stratification may have occurred. However, this explanation is rendered less likely by our inclusion of race as a covariate in the SNP-based analyses and the gene level analyses being limited to Caucasians.

## 5. Conclusions

Despite these limitations, the current study extends the previous research in a few ways. First, these findings provide initial suggestive evidence that those at greater genetic risk on *GAT1* are at increased risk for PTSD and MDD as well as PTSD and SUD, compared to just risk for PTSD alone. Therefore, those with a greater number of risk alleles on *GAT1* comprise a particularly risky group, on whom prevention and intervention efforts should be focused. Given the paucity of work examining the link between *GAT1* SNPs and these phenotypes, this work is incredibly important, as it provides initial insight into SNPs that may account for the shared genetic overlap in PTSD, MDD, and SUD.

## Figures and Tables

**Table 1:** Demographic and clinical characteristics of sample.

Variable	Control (*n* = 172)	PTSD (*n* = 314)	*p*
Age	43.2 ± 14.7	43.5 ± 14.4	0.820
CES	19.5 ± 8.7	21.7 ± 10.5	0.020
Race			0.001
CA	127 (26.1%)	207 (42.6%)	
AA	27 (5.6%)	91 (18.7%)	
Other	18 (3.7%)	16 (3.3%)	
Sex			0.056
Male	133 (27.4%)	266 (54.7%)	
Female	39 (8.0%)	48 (9.9%)	
Depression			<0.001
No depression	166 (34.2%)	111 (22.8%)	
Depression	6 (1.2%)	203 (41.8%)	
Substance Use Disorder			<0.001
No abuse	166 (34.2%)	215 (44.2%)	
Abuse	6 (1.2%)	99 (20.4%)	

Notes: For continuous variables, mean ± SD are shown. For categorical variables, *n* (%) are shown. Statistically significant associations (*p* < 0.05) are marked in red. PTSD = those with Posttraumatic Stress Disorder. CES = Combat Exposure Scale, CA = Caucasians, AA = African-Americans.

**Table 2:** List of SNPs with their genotype frequency, and *p*-values of testing for Hardy–Weinberg equilibrium by race.

SNP	Rare Homozygotes (%)	Heterozygotes (%)	Common Homozygotes (%)	Hardy–Weinberg *p*-Value			Genotype Frequency by Race *p*-Value
				CA	AA	Others	
rs2928078	33.7	50.0	16.3	0.377	0.289	0.504	0.002
rs2933308	44.2	42.4	13.4	0.559	1.000	0.478	0.008
rs720352	29.7	50.2	20.1	0.322	1.000	0.741	0.726
rs1919075	58.3	34.7	7.0	1.000	0.005	0.167	<0.001
rs2697150	58.0	35.2	6.8	0.753	0.188	1.000	0.086
rs2697134	74.2	24.4	1.4	0.006	0.203	0.042	0.014
rs11707097	73.0	23.9	3.2	0.821	0.340	0.113	0.343
rs2675163	62.2	33.8	4.0	0.766	0.690	1.000	0.001
rs2697149	57.6	35.5	6.9	0.643	0.261	1.000	0.183
rs1728816	41.3	43.8	14.9	0.243	1.000	0.722	0.699
rs2697153	51.6	38.0	10.4	0.481	0.143	0.298	<0.001
rs2944367	59.6	32.0	8.4	0.024	0.013	0.690	0.051
rs9876005	83.5	12.6	3.9	0.001	0.300	0.146	<0.001
rs9879137	78.0	18.1	3.9	0.103	0.406	0.371	<0.001
rs2272403	64.7	26.1	9.2	0.161	0.001	1.000	<0.001
rs11925331	78.0	18.5	3.5	0.059	0.270	0.371	<0.001

Notes: The *p*-values for comparing the genotype frequencies between African-Americans and Caucasians are also provided. CA and AA refer to Caucasians and African-Americans, respectively.

**Table 3:** The effect of each SNP on the risk of PTSD (i.e., individuals with only Posttraumatic Stress Disorder), PTSD-MDD (i.e., comorbid Posttraumatic Stress Disorder and Major Depressive Disorder), and PTSD-SUD (i.e., comorbid Posttraumatic Stress Disorder and Substance Use Disorder), adjusting for age, race, sex and CES.

SNP	PTSD				PTSD-MDD				PTSD-SUD			
	OR	95% Lower CI	95% Upper CI	*p*	OR	95% Lower CI	95% Upper CI	*p*	OR	95% Lower CI	95% Upper CI	*p*
rs2928078	1.28	0.96-	1.73	0.097	1.24	0.96	1.61	0.097	1.32	1.02	1.71	0.033
rs2933308	1.26	0.94	1.70	0.120	1.19	0.92	1.54	0.184	1.33	1.03	1.73	0.028
rs720352	1.35	1.01	1.81	0.045	1.17	0.91	1.51	0.211	1.27	0.99	1.63	0.064
rs1919075	0.65	0.47	0.89	0.008	0.71	0.53	0.94	0.018	0.66	0.49	0.89	0.006
rs2697150	0.92	0.67	1.28	0.625	0.92	0.70	1.22	0.570	0.88	0.66	1.16	0.347
rs2697134	0.80	0.53	1.23	0.306	0.73	0.50	1.06	0.096	0.76	0.52	1.11	0.158
rs11707097	1.08	0.74	1.61	0.692	1.05	0.75	1.46	0.797	1.00	0.72	1.40	0.993
rs2675163	1.22	0.86	1.75	0.268	1.28	0.93	1.75	0.127	1.10	0.81	1.51	0.540
rs2697149	1.02	0.74	1.43	0.895	1.01	0.76	1.34	0.960	0.97	0.73	1.28	0.824
rs1728816	0.95	0.71	1.26	0.717	0.95	0.74	1.22	0.708	0.94	0.73	1.21	0.651
rs2697153	1.08	0.79	1.47	0.637	1.02	0.78	1.33	0.904	1.10	0.84	1.44	0.503
rs2944367	0.89	0.65	1.20	0.433	0.91	0.70	1.19	0.485	0.90	0.68	1.18	0.427
rs9876005	1.27	0.76	2.20	0.369	1.22	0.81	1.85	0.348	1.07	0.72	1.59	0.729
rs9879137	1.01	0.64	1.59	0.982	1.11	0.76	1.62	0.587	0.93	0.65	1.33	0.682
rs2272403	1.45	1.01	2.10	0.046	1.44	1.07	1.95	0.018	1.16	0.87	1.55	0.304
rs11925331	1.37	0.87	2.22	0.183	1.31	0.90	1.91	0.156	1.05	0.73	1.50	0.803

Notes: The association between individual SNP and PTSD is based on logistic regression analysis. The association between individual SNP and PTSD-MDD or PTSD-SUD is based on proportional odds model. Statistically significant associations (*p* < 0.05) are marked in red.

**Table 4:** Loading of each SNP on the first four principal components used in the gene-level analysis.

	PC1	PC2	PC3	PC4
rs2928078	−0.511	0.052	0.100	0.133
rs2933308	−0.459	0.153	0.123	0.144
rs720352	−0.460	0.102	−0.019	0.086
rs1919075	0.393	0.124	−0.082	0.088
rs2697150	−0.051	0.347	−0.517	0.030
rs2697134	−0.086	−0.102	−0.017	−0.189
rs11707097	−0.285	−0.085	−0.027	−0.098
rs2675163	−0.003	−0.035	0.053	0.288
rs2697149	−0.042	0.349	−0.540	0.012
rs1728816	0.082	−0.300	0.147	0.576
rs2697153	−0.041	0.100	0.385	−0.560
rs2944367	−0.038	0.042	0.079	0.367
rs9876005	0.009	−0.413	−0.269	−0.069
rs9879137	−0.054	−0.474	−0.246	−0.074
rs2272403	−0.187	−0.332	−0.161	−0.143
rs11925331	−0.145	−0.281	−0.267	−0.077

Notes: Loadings of absolute values larger than 0.35 are marked in red.

**Table 5:** Gene-level association with PTSD (i.e., individuals with only Posttraumatic Stress Disorder), PTSD-MDD (i.e., comorbid Posttraumatic Stress Disorder and Major Depressive Disorder), and PTSD-SUD (i.e., comorbid Posttraumatic Stress Disorder and Substance Use Disorder), adjusting for age, sex, and CES in Caucasians.

	PTSD				PTSD-MDD				PTSD-SUD			
	OR	95% Lower CI	95% Upper CI	*p*-Value	OR	95% Lower CI	95% Upper CI	*p*-Value	OR	95% Lower CI	95% Upper CI	*p*-Value
Age	1.00	0.98	1.02	0.951	1.00	0.98	1.01	0.715	1.01	0.99	1.02	0.477
Female	0.79	0.43	1.44	0.429	0.81	0.47	1.39	0.440	0.64	0.37	1.09	0.101
CES	1.01	0.98	1.03	0.534	1.01	0.99	1.03	0.249	1.01	0.99	1.03	0.481
PC1	0.84	0.73	0.96	0.012	0.88	0.78	0.99	0.033	0.86	0.76	0.97	0.015

Notes: The gene-level association with PTSD is based on logistic regression analysis. The gene-level association with PTSD-MDD or PTSD-SUD is based on proportional odds model. Statistically significant associations (*p* < 0.05) are marked in red. OR refers to odds ratio.

## Appendix

**Table A1:** The effect of each SNP on the risk of PTSD (i.e., individuals with only Posttraumatic Stress Disorder), PTSD-MDD (i.e., comorbid Posttraumatic Stress Disorder and Major Depressive Disorder), and PTSD-SUD (i.e., comorbid Posttraumatic Stress Disorder and Substance Use Disorder) in Caucasians, adjusting for age, sex and CES.

SNP	PTSD				PTSD-MDD				PTSD-SUD			
	OR	95% Lower CI	95% Upper CI	*p*	OR	95% Lower CI	95% Upper CI	*p*	OR	95% Lower CI	95% Upper CI	*p*
rs2928078	1.31	0.94	1.84	0.12	1.20	0.89	1.62	0.23	1.35	1.00	1.83	0.05
rs2933308	1.16	0.84	1.63	0.38	1.12	0.83	1.51	0.47	1.32	0.98	1.78	0.07
rs720352	1.36	0.97	1.92	0.08	1.34	0.99	1.83	0.06	1.30	0.96	1.76	0.09
rs1919075	0.57	0.39	0.82	0.00	0.67	0.48	0.93	0.02	0.63	0.45	0.88	0.01
rs2697150	0.97	0.66	1.43	0.88	0.93	0.65	1.32	0.67	0.99	0.70	1.40	0.94
rs2697134	1.05	0.64	1.75	0.86	0.88	0.57	1.38	0.59	0.94	0.60	1.47	0.78
rs11707097	1.17	0.74	1.89	0.51	1.04	0.69	1.55	0.86	1.02	0.68	1.53	0.92
rs2675163	1.19	0.81	1.76	0.38	1.45	1.01	2.07	0.04	1.08	0.76	1.53	0.68
rs2697149	1.13	0.77	1.67	0.55	1.08	0.76	1.52	0.68	1.03	0.73	1.46	0.86
rs1728816	0.75	0.54	1.04	0.09	0.87	0.65	1.17	0.36	0.79	0.59	1.07	0.13
rs2697153	1.19	0.85	1.67	0.31	1.08	0.80	1.45	0.61	1.21	0.90	1.62	0.22
rs2944367	0.92	0.65	1.30	0.63	0.97	0.71	1.32	0.84	0.88	0.64	1.20	0.42
rs9876005	1.05	0.45	2.75	0.92	1.29	0.56	2.98	0.55	0.75	0.36	1.58	0.45
rs9879137	0.98	0.52	1.94	0.96	1.14	0.62	2.10	0.67	0.79	0.45	1.39	0.41
rs2272403	1.89	1.14	3.25	0.02	1.87	1.20	2.90	0.01	1.35	0.90	2.03	0.15
rs11925331	2.42	1.17	5.70	0.03	1.99	1.10	3.61	0.02	1.28	0.75	2.18	0.37

Notes: The association between individual SNPs and PTSD is based on logistic regression analysis. The association between individual SNP and PTSD-MDD or PTSD-SUD is based on proportional odds model. Statistically significant association (*p* < 0.05) is marked in red.

**Table A2:** The effect of each SNP on the risk of PTSD (i.e., individuals with only Posttraumatic Stress Disorder), PTSD-MDD (i.e., comorbid Posttraumatic Stress Disorder and Major Depressive Disorder), and PTSD-SUD (i.e., comorbid Posttraumatic Stress Disorder and Substance Use Disorder) in African-Americans, adjusting for age, sex and CES.

SNP	PTSD				PTSD-MDD				PTSD-SUD			
	OR	95% Lower CI	95% Upper CI	*p*	OR	95% Lower CI	95% Upper CI	*p*	OR	95% Lower CI	95% Upper CI	*p*
rs2928078	1.85	0.81	4.56	0.16	1.87	0.98	3.55	0.06	1.59	0.86	2.96	0.14
rs2933308	2.00	0.87	5.10	0.12	1.39	0.76	2.54	0.29	1.43	0.79	2.60	0.24
rs720352	1.06	0.51	2.24	0.87	0.63	0.37	1.08	0.10	1.05	0.62	1.77	0.85
rs1919075	1.99	0.61	11.89	0.33	1.12	0.53	2.40	0.76	1.00	0.48	2.07	1.00
rs2697150	1.15	0.54	2.60	0.73	1.26	0.72	2.19	0.42	0.76	0.45	1.28	0.30
rs2697134	0.39	0.12	1.18	0.10	0.40	0.16	1.00	0.05	0.39	0.15	1.01	0.05
rs11707097	0.95	0.39	2.33	0.90	1.09	0.54	2.20	0.81	0.95	0.48	1.87	0.88
rs2675163	0.71	0.23	2.36	0.55	0.51	0.23	1.17	0.11	0.63	0.27	1.47	0.28
rs2697149	1.07	0.48	2.60	0.87	1.16	0.64	2.10	0.63	0.94	0.54	1.66	0.84
rs1728816	1.61	0.76	3.66	0.23	0.87	0.51	1.47	0.60	1.35	0.77	2.34	0.29
rs2697153	0.71	0.26	2.13	0.51	0.86	0.39	1.92	0.71	0.81	0.37	1.76	0.59
rs2944367	0.47	0.21	1.06	0.07	0.56	0.30	1.05	0.07	0.61	0.32	1.16	0.13
rs9876005	1.18	0.57	2.56	0.66	1.02	0.60	1.72	0.95	1.22	0.73	2.06	0.45
rs9879137	0.82	0.39	1.74	0.60	0.95	0.56	1.61	0.84	1.01	0.59	1.70	0.99
rs2272403	0.82	0.43	1.53	0.53	0.94	0.59	1.51	0.81	0.82	0.52	1.30	0.40
rs11925331	0.84	0.40	1.80	0.65	0.98	0.56	1.72	0.96	0.81	0.47	1.39	0.44

Notes: For African Americans, only one SNP was found to be significant at a significance level of 0.05. Because African-Americans only made up 24.3% of the study cohort, the analyses on this small group of participants may have been under-powered and/or the estimates may not be stable or representative of the larger group of African-Americans. Statistically significant association (*p* < 0.05) is marked in red.

**Table A3:** Results using Genetic Risk Sum Score (GRSS).

	PTSD				PTSD-MDD				PTSD-SUD			
	OR	95% Lower CI	95% Upper CI	*p*-Value	OR	95% Lower CI	95% Upper CI	*p*-Value	OR	95% Lower CI	95% Upper CI	*p*-Value
Age	1.00	0.98	1.02	0.943	1.00	0.98	1.01	0.760	1.01	0.99	1.02	0.465
Female	0.78	0.43	1.44	0.426	0.79	0.46	1.36	0.393	0.63	0.37	1.08	0.097
CES	1.01	0.98	1.03	0.576	1.01	0.99	1.03	0.319	1.01	0.99	1.03	0.493
GRSS	2.50	1.46	4.39	0.001	2.19	1.36	3.55	0.001	1.65	1.13	2.42	0.009

Notes: The gene-level association with PTSD (i.e., those with only Posttraumatic Stress Disorder) is based on logistic regression analysis. The gene-level association with PTSD-MDD (i.e., comorbid PTSD-Major Depression) or PTSD-SUD (i.e., comorbid PTSD-Substance Use Disorder) is based on proportional odds model. CES = Combat Exposure Scale. Statistically significant association (*p* < 0.05) is marked in red.
